# The relationship between the pattern of shift work and sleep disturbances in Korean firefighters

**DOI:** 10.1007/s00420-019-01496-3

**Published:** 2019-11-25

**Authors:** Tae-Won Jang, Kyoung Sook Jeong, Yeon-Soon Ahn, Kyeong-Sook Choi

**Affiliations:** 1 Department of Occupational and Environmental Medicine, College of Medicine, Hanynag University, 222, Wangsimni-ro, Seongdong-gu, Seoul, 04763 Republic of Korea; 2 Department of Occupational and Environmental Medicine, Hallym Sacred Heart Hospital, Hallym University College of Medicine, Anyang, Republic of Korea; 3 Department of Preventive Medicine, Yonsei University Wonju College of Medicine, Wonju, Republic of Korea; 4 Department of Neuropsychiatry, Eulji University Hospital, Daejeon, Republic of Korea

**Keywords:** Firefighters, Insomnia, Shift work, Sleep disturbance

## Abstract

**Purpose:**

Sleep disturbances are prevalent in firefighters, but the relationship between patterns of shift work and sleep disturbances has not yet been investigated. Here, this relationship has been evaluated in Korean firefighters.

**Methods:**

A cross-sectional study was conducted using an online questionnaire, which captured demographic, psychosocial and work-related characteristics. Sleep disturbance was assessed using the insomnia severity index (ISI). The relationship between insomnia and work-related factors (including type of shift work and the frequency of emergency events and off-duty work which means overtime work on off days) was analyzed.

**Results:**

A total of 9810 firefighters completed the survey, representing approximately 21.5% of all Korean firefighters; data from 9738 subjects were included in the analysis. All firefighter roles were significantly associated with insomnia; the odds ratio (OR) was 2.456 (95% confidence interval [CI] 1.461–4.128) for fire suppression and 1.871 (95% CI 1.105–3.167) for the emergency medical services and rescue. However, the pattern of shift work did not show a statistically significant relationship. The OR increased along with the frequency of emergency events and off-duty work (*p* value for trend < 0.05).

**Conclusions:**

This study found a significant association between the frequency of emergency and off-duty work and insomnia in Korean firefighters, whereas the pattern of shift work showed no significant relationship. Therefore, measures to reduce the frequency of emergency and off-duty work are required to prevent sleep disturbances in firefighters.

## Introduction

Firefighters are responsible for the safety of the public and the service is required 24 h a day. To meet these requirements, it is necessary to perform shift work and an elevated risk of sleep disturbances has been reported in firefighters. The prevalence of sleep disturbances in firefighters has been reported to range from 13.7 to 73%, with the differences arising from the variation in assessment tools used. Haddock et al. (2013) assessed daytime sleepiness among firefighters in the USA using the Epworth sleepiness scale (ESS) and reported that the prevalence of excessive daytime sleepiness was 13.7–14.0%. Lim et al. (2014) investigated sleep disturbances in firefighters using the Pittsburgh sleep quality index (PSQI) and reported the prevalence of sleep disturbances to be 48.7%. Barger et al. (2015) investigated sleep disturbance in firefighters using the Berlin Questionnaire, Athens Insomnia Scale and the Restless Legs Syndrome Epidemiology, Symptoms and Treatment Questionnaire, reporting the prevalence of sleep disorders (including obstructive sleep apnea, insomnia, shift work disorder and restless leg syndrome) to be 37.2%. Billings and Focht (2016) investigated the sleep quality in firefighters using the PSQI and reported that 73% of firefighters had poor sleep quality.

Rotating shift work is classified as being fast (changing shift every 2–3 days), slow (changing shift every 3–4 weeks), or a permanent night shift. Some researchers have reported that permanent night shifts might be better for workers’ health and sleep (Karhula et al. 2018; Pilcher et al. 2000), whereas others have suggested that permanent night shifts should be avoided because of sleep deprivation (Folkard 2008; Knauth and Hornberger 2003). Although some researchers have shown that permanent night shifts can be advantageous, guidelines for shift workers recommend that permanent night shifts should be avoided as most workers never really adapt to the schedule (Rosa and Coligan 1997; Health and Safety Executives 2006). Regarding the fast and slow shift rotations, there is no consensus which is preferable with respect to workers’ health. Some researchers suggest that fast rotations are preferable as they minimize continuous night shifts, which induce sleep loss and fatigue (Folkard 2008; Karhula et al. 2016; Knauth and Hornberger 2003). However, others have reported that slow rotating shifts are preferable as they allow sufficient time for workers to adapt to night shifts and subjects reported better sleep quality and well-being (Dahlgren 1981; Totterdell et al. 1995a, b; Chang et al. 2011). Regarding the direction of shift rotations, these are classified as forward or clockwise (morning to afternoon to night shift) or backward or counterclockwise (morning to night to afternoon shift). Although there has been some debate about the speed of shift rotation, most researchers are in agreement that a forward rotation is preferable to the backwards direction (Knauth and Hornberger 2003; Shiffer et al. 2018; Viitasalo et al. 2008).

Although sleep disturbances in firefighters are well documented, the relationship between the pattern of shift work and sleep disturbances has not yet been investigated. Therefore, this study aimed to investigate this relationship in Korean firefighters.

## Subjects and methods

### Subjects

This was a cross-sectional study, which was performed using an online questionnaire. A web page was constructed and the instructions for the online survey were distributed to all Korean firefighters. Subjects were initially required to enter their e-mail address and password (to avoid duplicate responses), followed by personal information, which was not included in the survey. The survey was conducted between November 2017 and August 2018, and was approved by the Institutional Review Board of Yonsei University Wonju Severance Christian Hospital (CR318031).

### Variables for analysis

The questionnaire captured demographic, psychosocial and work-related characteristics. Demographic characteristics included age, sex, height, weight, education, income, marital status, smoking status, alcohol consumption, caffeine intake and exercise. Psychosocial characteristics included insomnia, fatigue, depression, anxiety, and post-traumatic stress disorder (PTSD). The Korean version of the Insomnia Severity Index (ISI) was used to assess the severity of insomnia (Cho et al. 2014). The ISI consists of seven items assessing the symptoms of insomnia during the previous 2 weeks. The ISI score ranges from 0 to 28, classifying insomnia as none (0–7), mild (8–14), moderate (15–21) or severe (22–28) (Bastien et al. 2001).

Fatigue and psychiatric conditions including depression, anxiety, and post-traumatic stress disorder (PTSD) were reported to be associated with sleep disturbances (Germain et al. 2017; Gould et al. 2018; Steiger and Pawlowski 2019; Wendt et al. 2019). We assessed fatigue, fatigue, anxiety, and PTSD to control the effect of those comorbidities on sleep disturbance. Fatigue was assessed the Fatigue Severity Scale (FSS), which was reported to be a reliable instrument to quantify fatigue (Valko et al. 2008). This tool consists of nine items, with a score range of 9–63; the Korean version of the FSS was used to assess fatigue in the study subjects (Lee et al. 2013). Psychiatric comorbidities were evaluated using the Patient Health Questionnaire-9 (PHQ-9), Generalized Anxiety Disorder-7 (GAD-7) and Primary Care post-traumatic stress disorder screen for DSM-5 (PC-PTSD). The PHQ-9 includes nine items that assess depressive symptoms in the previous 2 weeks, with a score range of 0–27 that classifies depression as none or minimal (0–4), mild (5–9), moderate (10–14), moderately severe (15–19), and severe (20–27) (Kroenke et al. 2001). The GAD-7 includes seven items that determine anxiety symptoms in the previous 2 weeks; scores range from 0 to 21 and classify anxiety as none or minimal (0–4), mild (5–9), moderate (10–14), and severe (15–21) (Spitzer et al. 2006). The PC-PTSD includes five items to identify individuals with probable PTSD and has been reported to be a reasonable screening tool for PTSD in primary care clinics or the community setting (Spoont et al. 2015). The scores range from 0 to 5 defining subjects as being either normal (0–2) or having probable PTSD (3–5) (Prins et al. 2004).

**Fig. 1:**
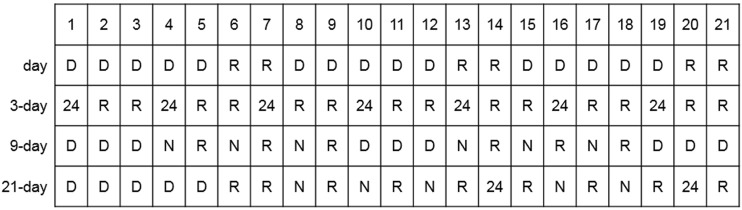
Shift work schedule for Korean firefighters. The numbers (1–21) represent the date; day = day work; 3-day = 3-day cycle; 9-day = 9-day cycle; 21-day = 21-day cycle; D = day work; R = rest day; 24 = 24 h shift; N = night shift

Work-related characteristics included the job type, employment period, work schedule, and the frequency of emergencies and off-duty work (overtime work on off-duty days). Work schedule was classified as day work, 3-, 9- and 21-day cycle, as shown in Fig. 1. The 3-day cycle consists of one 24-h shift followed by 2 rest days. The 9-day cycle consists of three daytime shifts followed by three 12-h night shifts and each night shift is followed by 1 rest day. In the 21-day cycle, the first week consists of five daytime shifts followed by 2 rest days; the second week consists of 12-h night shifts alternating with a rest day until day 14, which is a 24-h shift; the third week starts with a rest day followed by two 12-h night shifts interspersed with rest days. On day 20, the firefighter works a 24-h shift. The last day is a rest day. Daytime shift was from 9:00 a.m. to 6:00 p.m., and night shift was from 6:00 p.m. to 9:00 a.m. the next day.

### Statistical analysis

Age was categorized into three groups (< 40, 40–49, and ≥ 50 years) and body mass index (BMI) into two (< 25 and ≥ 25 kg/m^2^). Education was grouped as high school, college, university or graduate school. Monthly income was grouped as < 3000, 3000–4999, and ≥ 5000 (the unit being × 1000 KRW). Marital status was captured as married, unmarried, and divorce or bereaved. Smoking statues was described as either never/ex-smoker or smoker. Subjects who drank more than seven cups of Soju (most common Korean liquor) per week were considered to be moderate or heavy drinkers. Caffeine intake was assessed with the frequency of coffee or tea intake. The amount of weekly exercise was also captured.

Sleep patterns were categorized as normal (ISI score < 15) or insomnia (ISI score ≥ 15). Fatigue was considered to be absent (FSS score < 36) or present (FSS score ≥ 36), as was depression (PHQ-9 score < 10 or ≥ 10, respectively), anxiety (GAD-7 score < 10 or ≥ 10, respectively) and PTSD (PC-PTSD score < 3 or ≥ 3, respectively).

Four job types were captured: administrative (reference), fire suppression, emergency medical service (EMS) or rescue, and others (such as fire investigation and fire engine or ambulance driving). Fire suppression is a job that extinguishes fire, EMS is a job that carries emergent patients by an ambulance, and rescue is a job that rescues people in the event of an accident. Others included fire investigation (a job that investigate the cause of the fire), drivers of fire engine and ambulance, and so on. Work schedule was classified as day work (reference), 3-day, 6-day, 21-day cycle, and others. The employment period was described as < 5, 5–9, 10–14, and ≥ 15 years. The frequency of emergency events was evaluated in five groups (< 5 (reference), 5–9, 10–19, 20–39, and ≥ 40 times/week). The frequency of off-duty work was classified as < 1 (reference), 1–2, 3–4, and ≥ 5 times/month.

Subjects with an ISI score < 15 comprised the control group and subjects with an ISI score ≥ 15 comprised the insomnia group. Chi square tests were performed to compare each variable in the normal and insomnia groups. Logistic regression analyses were performed to investigate the relationship between work-related variables and insomnia. The dependent variable was insomnia and independent variables were demographic, psychosocial, and work-related variables. Model 1 was adjusted for age and sex, model 2 was adjusted for demographic and psychosocial characteristics, and model 3 was adjusted for demographic, psychosocial, and work-related characteristics.

Statistical analyses were performed using SAS windows version 9.4 (SAS Institute Inc., Cary, NC, USA). *P* values of < 0.05 were considered to indicate statistically significant differences.

## Results

A total of 9810 firefighters completed the survey, which represents approximately 21.5% of all Korean firefighters. Subjects who did not complete the demographics (*n* = 50) and work-related (*n* = 22) features were excluded from the analyses. Therefore, data from a total of 9738 subjects were included in the study. Among them, the prevalence of moderate-to-severe insomnia (ISI ≥ 15) was 9.1% (883 among 9738), and the prevalence of mild-to-severe insomnia (ISI ≥ 8) was 41.8% (4069 among 9738).

Table 1 shows the demographic and psychosocial characteristics of the control and insomnia groups. The age and sex distribution differed significantly between the two groups, with the control group being younger and the insomnia group having a higher proportion of female subjects (both *p* < 0.05). No significant differences were seen in terms of BMI, education, or exercise. There were more subjects classified as high income, married, divorced or bereaved, never or ex-smoker, and moderate or heavy drinkers in the insomnia group (*p* < 0.05). Fatigue, depression, anxiety, and PTSD were more common in the insomnia group (*p* < 0.05).

**Table 1:** Demographic and psychosocial characteristics of the study subjects

Variables	Category	Control *n* = 8855	Insomnia *n* = 883
Age, years*	< 40	4838 (54.6)	428 (48.5)
	40–49	2585 (29.2)	273 (30.9)
	≥ 50	1432 (16.2)	182 (20.6)
Sex*	Male	8273 (93.4)	807 (91.4)
	Female	582 (6.6)	76 (8.6)
Body mass index, kg/m^2^	< 25	5628 (63.6)	574 (65.0)
	≥ 25	3227 (36.4)	309 (35.0)
Education, years	High school	1770 (20.0)	174 (19.7)
	College	2886 (32.6)	269 (30.5)
	University	4042 (45.6)	414 (46.9)
	Graduate school	157 (1.8)	26 (2.9)
Income, × 1000 KRW/month*	< 3000	3226 (36.4)	266 (30.1)
	3000–4999	4291 (48.5)	460 (52.1)
	≥ 5000	1338 (15.1)	157 (17.8)
Marital status*	Married	6178 (69.8)	652 (73.8)
	Unmarried	2551 (28.8)	208 (23.6)
	Divorced or bereaved	126 (1.4)	23 (2.6)
Smoking*	Never or ex-smoker	6313 (71.3)	663 (75.1)
	Current smoker	2542 (28.7)	220 (24.9)
Alcohol consumption*	None or social	6099 (68.9)	576 (65.2)
	Moderate or heavy^a^	2756 (31.1)	307 (34.7)
Caffeine intake, cups/day*^b^	< 1	1496 (16.9)	182 (20.6)
	1–2	4377 (49.4)	385 (43.6)
	≥ 3	2982 (33.7)	316 (35.8)
Exercise, times/week	0–1	3256 (36.8)	351 (39.8)
	2–3	3339 (37.7)	309 (35.0)
	≥ 4	2260 (25.5)	223 (25.2)
Fatigue*	No	5685 (64.2)	162 (18.3)
	Yes	3170 (35.8)	721 (81.7)
Depression*	No	8697 (98.2)	663 (75.1)
	Yes	158 (1.8)	220 (24.9)
Anxiety*	No	8744 (99.0)	705 (82.4)
	Yes	92 (1.0)	151 (17.6)
Post-traumatic stress disorder*	No	8222 (92.9)	563 (63.8)
	Yes	633 (7.1)	320 (36.2)

The data are presented as number (%)

*p < 0.05

^a^More than seven cups of Soju per week

^b^Frequency of coffee or tea intake

Table 2 shows the work-related characteristics among the normal and insomnia groups. The insomnia group included a higher proportion of subjects in the role of fire suppression and a lower proportion of subjects in administrative roles; the employment period was also longer in the insomnia group (*p* < 0.05). The work schedule did not differ significantly between the two groups (*p* > 0.05). The frequency of emergency events and off-duty work was higher in the insomnia group (*p* < 0.05).

**Table 2:** Work-related characteristics of the study subjects

Variables	Category	Control *n* = 8855	Insomnia *n* = 883
Type of job*	Administrative	774 (8.7)	50 (5.7)
	Fire suppression	2822 (31.9)	308 (34.9)
	EMS/rescue	3185 (36.0)	318 (36.0)
	Others	2074 (23.4)	207 (23.4)
Employment period, years*	< 5	2914 (43.1)	230 (36.6)
	5–9	2014 (29.8)	186 (29.6)
	10–14	1254 (18.5)	141 (22.4)
	≥ 15	584 (8.6)	72 (11.5)
Work schedule	Day work	921 (10.4)	77 (8.7)
	3-day cycle	621 (7.0)	55 (6.2)
	9-day cycle	1069 (12.1)	119 (13.5)
	21-day cycle	5758 (65.0)	576 (65.2)
	Others	486 (5.5)	56 (6.3)
Frequency of emergency events, times per week*	< 5	2694 (30.4)	183 (20.7)
	5–9	2094 (23.6)	188 (21.3)
	10–19	2269 (25.6)	254 (28.8)
	20–39	1452 (16.5)	202 (22.9)
	≥ 40	336 (3.8)	56 (6.3)
Frequency of off-duty work, times per month*	< 1	4097 (46.3)	307 (34.8)
	1–2	3796 (42.9)	423 (47.9)
	3–4	718 (8.1)	108 (12.2)
	≥ 5	244 (2.8)	45 (5.1)

EMS means emergency medical service

*p < 0.05

Table 3 shows the factors associated with insomnia. All jobs, other than administrative, were significantly associated with insomnia in all models: the odds ratio (OR) in model 3 was 2.456 (95% confidence interval (CI) 1.461–4.128) in fire suppression, 1.871 (95% CI 1.105–3.167) in EMS and rescue, and 1.968 (95% CI 1.163–3.331) in other jobs. Shift patterns showed no statistically significant differences in all models. Regarding the frequency of emergency events and off-duty work, the OR increased with the frequency of both (*p* value for trend < 0.05).

**Table 3:** Relationship between work-related factors and insomnia

Variables	Model 1	Model 2	Model 3
Type of job			
Administrative	(Reference)	(Reference)	(Reference)
Fire suppression	1.725 (1.264–2.354)	2.176 (1.500–3.158)	2.456 (1.461–4.128)
EMS/rescue	1.735 (1.270–2.371)	2.093 (1.443–3.036)	1.871 (1.105–3.167)
Others	1.583 (1.148–2.182)	1.844 (1.258–2.703)	1.968 (1.163–3.331)
Work schedule			
Day work	(Reference)	(Reference)	(Reference)
3-day cycle	1.177 (0.818–1.692)	1.388 (0.906–2.128)	0.847 (0.500–1.434)
9-day cycle	1.448 (1.071–1.958)	1.444 (1.010–2.064)	0.858 (0.536–1.374)
21-day cycle	1.338 (1.041–1.721)	1.513 (1.122–2.038)	0.802 (0.524–1.227)
Others	1.530 (1.064–2.202)	1.618 (1.052–2.487)	0.892 (0.528–1.506)
Frequency of emergency events, times per week*			
< 5	(Reference)	(Reference)	(Reference)
5–9	1.357 (1.098–1.678)	1.345 (1.056–1.715)	1.231 (0.960–1.580)
10–19	1.708 (1.400–2.083)	1.571 (1.248–1.978)	1.453 (1.142–1.848)
20–39	2.173 (1.757–2.687)	1.896 (1.479–2.431)	1.816 (1.388–2.377)
≥ 40	2.767 (1.999–3.829)	2.421 (1.660–3.532)	2.340 (1.570–3.486)
Frequency of off-duty work, times per month*			
< 1	(Reference)	(Reference)	(Reference)
1–2	1.555 (1.333–1.815)	1.307 (1.093–1.562)	1.252 (1.042–1.504)
3–4	2.135 (1.688–2.700)	1.540 (1.172–2.024)	1.477 (1.115–1.955)
≥ 5	2.569 (1.828–3.609)	1.421 (0.938–2.151)	1.563 (1.022–2.390)

The data are presented as odds ratios (95% confidence interval). EMS means emergency medical service. Model 1 adjusts for age and sex; model 2 adjusts for demographic characteristics (age, sex, BMI, education, income, marital status, smoking, alcohol consumption, caffeine intake, and exercise), and comorbidities (fatigue, depression, anxiety, and PTSD); model 3 adjusts for the demographic characteristics, comorbidities, and work-related characteristics (type of job, employment period, work schedule, frequency of emergency events and off-duty work). *p-value for trend < 0.05 in model 3

## Discussion

This study has demonstrated that the type of job, the frequency of emergency events and off-duty work show a positive association with insomnia, and that the strength of this relationship increased along with the frequency of emergency events and off-duty work. No significant relationship was found between insomnia and shift patterns.

Most Korean firefighters undertake a variety of rotating shift work, but it is difficult to distinguish them as fast or slow as they perform a night shift every other day and do not perform continuous night shifts. In addition, Korean firefighters’ shift patterns are neither a forward nor backward rotation. Therefore, it is not possible to evaluate Korean firefighters’ shift work in terms of the speed and direction of rotations.

There are some features associated with shift work including the speed and direction of shift rotations, the length of the recovery periods between shifts, the length of the shifts, and the timing of shift change. From these points of view, there are some differences among the shift patterns of Korean firefighters. As to the speed of rotation, one shift of 3-day cycle was 1 day so 3-day cycle is fast rotation. Whereas 9-day and 21-day cycle, the duration of night shift was 6 days (9-day cycle) and 14 days (21-day cycle), so 9-day and 21-day cycle cannot be classified as fast rotation nor slow rotation. As to the length of the recovery periods between shifts, the recovery period of 3-day cycle was 48 h. In the 9-day cycle, it ranged from 24 h (between night shift and day shift) to 48 h (between day shift and night shift). In the 21-day cycle, it ranged from 24 h (between night shift and 24-h shift) and 72 h (between day shift and night shift). Other features were not different from each other: the length of shifts was 8 h (day shift), 15 h (night shift), and 24 h (24-h shift); the timing of shift change was 9:00 a.m. and 6:00 p.m.

In the logistic regression analyses, 9-day and 21-day cycle was significantly associated with insomnia in the model 1 and 2, whereas 3-day cycle was not significant. As discussed earlier, 3-day cycle is fast rotation but 9-day and 21-day cycles were not. Although there has been controversy in the effect of fast and slow rotation, most guides for shift work recommended fast rotation (change shift every 2–3 days) or slow rotation (change shift every 3–4 weeks), and avoid weekly rotation (change every 1 week) which was the worst shift pattern (Rosa and Coligan 1997; Health and Safety Executives 2006). This could explain the results that 3-day cycle was not significant in the statistical analyses. In addition, the recovery period of 9-day and 21-day cycle was same or shorter than 3-day cycle (except 72 h from day shift and night shift in 21-day cycle). So recovery period shorter than 48 h might not be enough to relieve fatigue in firefighters.

However, shift patterns were not significant associated with insomnia after adjusting work-related characteristics in the model 3. Type of job, the frequency of emergency events and off-duty work were significant variables in all three models. This means that shift patterns were less important factors on sleep disturbances compared as with the type of job and the frequency of emergency events and off-duty work.

The role of a firefighter is very physically demanding. The US Department of Labor (1991) outlines five levels of physical demand: sedentary, light, medium, hard and very hard work. According to this classification, the physical demands of the firefighter role are determined to be ‘very hard’ (US Department of Labor 2015). According to the study by Ainsworth et al. (2011), the energy requirement for the task of ‘victim rescue’ and ‘automobile accident’ was 6.8 MET, and the energy requirement for the task of ‘fire suppression’ was 8.0 MET. High metabolic equivalent of task (MET) means high intensity of physical activity, so those tasks of firefighters are very high physical intensity. The OR of fire suppression (2.456, 95% CI 1.461–4.128) was higher than EMS/rescue (1.871, 95% CI 1.105–3.167) and others (1.968, 95% CI 1.163–3.331) in the model 3. This finding may be resulted from the higher physical demand of fire suppression (8.0 MET) as compared with rescue or automobile accident (6.8 MET).

Sleep disturbances may be related to the duration of off-duty time, which may explain the differences seen in previous studies of shift length and sleep quality. Researchers have reported that an increase in off-duty days can improve sleep duration and quality and that the duration of off-duty time is dependent on the workload and shift length (Totterdell et al. 1995a, b; Åkerstedt and Kecklund 2017). In addition, Rupp et al. (2009) reported that long sleep duration for several days before several days of sleep restriction improved the recovery or alterness and performance. According to these studies, one off-duty day is not sufficient and two or more days are required after shift work, which disrupts circadian rhythms (Totterdell et al. 1995a, b; Åkerstedt and Kecklund 2017). Among the shift work schedule of Korean firefighters, 3-day cycle includes 2 off-duty days, whereas 9- and 21-day cycles include only one off-duty day between night or 24-h shifts. Based on the observations of previous studies, the shift pattern of Korean firefighters is not desirable due to the long shift length and insufficient number of off-duty days.

In the absence of an emergency call out, it is possible for firefighters to sleep during a night shift. However, most firefighters are required to respond to emergencies during night shifts, which results in sleep deprivation. In addition, to respond to a major fire or accident, firefighters will be required to work on their off-duty days, which were defined as off-duty work. In the current study, 54.8% of Korean firefighters reported that they had undertaken off-duty work more than once a month. In the multiple logistic regression analyses, the frequency of emergency events and off-duty work was significantly related to insomnia, whereas the shift work schedule was not. Emergencies during night shifts and on off-duty days may, therefore, result in sleep deprivation and insufficient rest, which could account for the results seen in our study. This finding supports previous data demonstrating the relationship between the duration of off-duty time and sleep disturbances in firefighters.

This study has several limitations. First, the study design was cross sectional, meaning that the temporal relationship between insomnia and related factors could not be evaluated. Secondly, as the information was derived from an online questionnaire, the potential influence of recall bias cannot be excluded. In addition, there might be a selection bias because a proper randomization was not achieved. Despite these limitations, this study has strength that we investigated the characteristics of shift work: shift work schedule, shift length, and the duration of off-duty. In addition, the response rate for online questionnaire was so good (21.5%), which means large population over 20% of all Korean firefighters was included in this study, thanks to the good response rate.

## Conclusions

This study found that the frequency of emergency and off-duty work was significantly related to insomnia in Korean firefighters, whereas the pattern of shift work was not. Therefore, measures to prevent sleep disturbances in firefighters would need to include a reduction in the requirement for emergency and off-duty work. The findings of this study may be helpful in the future design of shift work patterns for firefighters.
